# Objective and subjective measurement of sedentary behavior in human adults: A toolkit

**DOI:** 10.1002/ajhb.23546

**Published:** 2020-12-05

**Authors:** Justin Aunger, Janelle Wagnild

**Affiliations:** ^1^ Health Services Management Centre Park House, University of Birmingham England UK; ^2^ Department of Anthropology Durham University Durham England UK

## Abstract

**Objectives:**

Objectives: Human biologists are increasingly interested in measuring and comparing physical activities in different societies. Sedentary behavior, which refers to time spent sitting or lying down while awake, is a large component of daily 24 hours movement patterns in humans and has been linked to poor health outcomes such as risk of all‐cause and cardiovascular mortality, independently of physical activity. As such, it is important for researchers, with the aim of measuring human movement patterns, to most effectively use resources available to them to capture sedentary behavior.

**Methods:**

This toolkit outlines objective (device‐based) and subjective (self‐report) methods for measuring sedentary behavior in free‐living contexts, the benefits and drawbacks to each, as well as novel options for combined use to maximize scientific rigor. Throughout this toolkit, emphasis is placed on considerations for the use of these methods in various field conditions and in varying cultural contexts.

**Results:**

Objective measures such as inclinometers are the gold‐standard for measuring total sedentary time but they typically cannot capture contextual information or determine which specific behaviors are taking place. Subjective measures such as questionnaires and 24 hours‐recall methods can provide measurements of time spent in specific sedentary behaviors but are subject to measurement error and response bias.

**Conclusions:**

We recommend that researchers use the method(s) that suit the research question; inclinometers are recommended for the measurement of total sedentary time, while self‐report methods are recommended for measuring time spent in particular contexts of sedentary behavior.

## 1 INTRODUCTION

Biological anthropologists and human biologists are increasingly interested in applying an evolutionary perspective to understand and potentially mitigate the effects of modern, industrialized lifestyles on health. While there are limits to the extent to which the activity patterns of contemporary hunter‐gatherer populations can be extrapolated to infer our evolutionary past (Cordain, Gotschall, Eaton, & Eaton, 1998), there is a general consensus that human physiology evolved within an ecological context which was characterized by a higher level of physical activity than is usually observed among contemporary industrialized populations (Cordain et al., 1998; Malina & Little, 2008). The differences in physical activity levels between hunter‐gatherer and contemporary industrialized human populations have been widely recognized (Eaton & Eaton, 2003; Malina & Little, 2008; Pontzer, Wood, & Raichlen, 2018). Compared to many industrialized populations, contemporary hunter‐gatherer populations have higher (though variable) levels of physical activity (Jenike, 2001), involving traveling long distances, often while carrying heavy loads, in the pursuit of hunted prey and foraged food as well as the relocation of camps (Bentley, 1985; Hilton & Greaves, 2008; Hurtado, Hawkes, Hill, & Kaplan, 1985; Marlowe, 2005; Odea, 1991). The physical activity patterns of urban populations are comparatively very low (Raichlen et al., 2017; Tucker, Welk, & Beyler, 2011), and this “mismatch” in physical activity levels has been implicated as a key contributor to the recent rise of cardiometabolic diseases (Eaton & Eaton, 2003; Raichlen et al., 2017).

While the effects of physical inactivity are well‐understood within this paradigm, the role of sedentary time has been less appreciated. Epidemiological evidence indicates that sedentary time, defined by the Sedentary Behavior Research Network as time spent sitting or lying down with low energy expenditure (<1.5 metabolic equivalents [METs]) during waking hours (Sedentary Behavior Research Network, 2012), is associated with adverse health outcomes including all‐cause mortality, cardiovascular disease, and incidence of type 2 diabetes (Ekelund et al., 2019; Patterson et al., 2018). It is important to note that the effect of sedentary time persists even after time spent in moderate‐to‐vigorous physical activity is taken into account (Ekelund et al., 2019; Patterson et al., 2018). One of the key mechanisms by which sedentary time impacts health is thought to be muscle inactivity. Periods of sitting or lying down require little to no muscle contraction (Tikkanen et al., 2013) and muscle inactivity has been shown to alter the expression of genes involved in carbohydrate and lipid metabolism (Hamilton, Hamilton, & Zderic, 2007; Latouche et al., 2013). Other non‐ambulatory postures such as standing and squatting recruit comparatively more muscle groups (Gao et al., 2017; Tikkanen et al., 2013), and experimental evidence suggests that this level of muscle activity is linked to better biomarker profiles compared to passive sitting (Gao et al., 2017; Thorp et al., 2014). Thus, a seated or reclined posture during waking hours is a key distinguishing feature of sedentary time.

Broadly speaking, while, in recent decades, sedentary time is often discussed as a byproduct of technological developments (Ng & Popkin, 2012), it is not necessarily an evolutionary novelty. Time‐use data has referenced contemporary hunter‐gatherer populations spending substantial amounts of time resting, sitting, and relaxing during waking hours (Dyble, Thorley, Page, Smith, & Migliano, 2019; Hill, Kaplan, Hawkes, & Hurtado, 1985; Hurtado et al., 1985). One study has objectively measured sedentary time among hunter‐gatherers, the Hadza, reporting that they accumulate 9.8 hours of sedentary time per day (Raichlen et al., 2020)—a figure similar to measurements using comparable methods in countries such as the United States (9.1 hours (Craft et al., 2012), UK (9.1 hours (Edwardson et al., 2020), and Australia (8.8 hours [Bellettiere et al., 2017]). However, this study identified that approximately 30% of time is spent in non‐ambulatory postures (eg, squatting, kneeling) which require more muscular activation than the kinds of passive sitting common in industrialized contexts (Raichlen et al., 2020). This raises key questions about how the nature of sedentary time might differ between populations and whether the accumulation of sedentary time in a passive seated or reclined posture represents another form of “inactivity mismatch.”

Within northern, industrialized settings, the manner in which sedentary time is accumulated may also be important. Epidemiological and experimental evidence suggests that breaking up periods of prolonged sitting is linked to improved cardiometabolic biomarker profiles (Bellettiere et al., 2017; Carson et al., 2014; Dunstan et al., 2012; Healy et al., 2008; Wagnild, Hinshaw, & Pollard, 2019). Additionally, “sit‐to‐stand transitions,” or the process of going from sitting to standing, are often a key target of interventions to reduce sedentary behavior due to their association with physical function in older people and the mobility impaired. As such, it can be important for studies to be able to capture these transitional movements (Keevil et al., 2016). There is also a large body of evidence suggesting that the context in which sedentary behavior occurs might also be key. For example, time spent watching television, which is often assumed to take place while sitting or lying down, is consistently more strongly associated with poor health outcomes than total sedentary time (Maher, Mire, Harrington, Staiano, & Katzmarzyk, 2013; Stamatakis, Hamer, Tilling, & Lawlor, 2012; Wagnild et al., 2019). Understanding time spent in specific sedentary behavior contexts is, therefore, often of interest, particularly to inform interventions and public health guidelines (Troiano, Gabriel, Welk, Owen, & Sternfeld, 2012). Similarly, evidence suggests that certain sub‐populations who are already highly sedentary and highly physically inactive, such as older adults, are at greater risk from the health impact of engaging in additional sedentary behavior (Aunger, Doody, & Greig, 2018; Aunger, Greaves, Davis, & Greig, 2019). It is, therefore, valuable to measure time spent in particular sedentary behavior contexts, as well as to measure patterns of sedentary time accumulation, while considering different subpopulations, to understand how time spent in sedentary behaviors impacts health.

This article, within the context of the American Journal of Human Biology toolkit series, highlights methods available to human biologists for measuring sedentary time and sedentary behavior in free‐living settings. We discuss objective measures of sedentary time, which are best suited for measuring total sedentary time and patterns of sedentary time, as well as subjective methods, which are important for determining the context of sedentary behavior. Throughout our discussion, we place particular emphasis on the balance between the practicalities of using each method in the field (eg, participant compliance, costs and burden to the researchers, language, or cultural considerations) and the validity of method (ie, how well the method measures what it purports to measure). In doing so, we hope to provide a comprehensive resource to those interested in measuring sedentary behavior.

## 2 OBJECTIVE MEASUREMENTS OF SEDENTARY TIME

Table 1 lists the strengths and weaknesses of the methods outlined below.

**TABLE 1:** Considerations for methods of measuring sedentary behavior

Measurement method	Validity	Reliability	Advantages	Disadvantages	Financial cost	Burden (researcher)	Burden (participant)	Considerations for field use	References for validation and for further reading
Direct observation	Very high (as long as the observer(s) is/are adequately trained to classify behaviors)	Very high (as long as the observer(s) is/are adequately trained to classify behaviors)	Allows for distinguishing between non‐ambulatory postures, allows assessment of context, requires minimal equipment	Extremely high burden on both researcher and participant, possibility that participant may modify behavior due to being directly observed	Low in terms of equipment but high in terms of researcher time	Very high	Very high	Can distinguish between postures such as ground‐sitting, chair‐sitting, squatting, and kneeling which may be particularly important in non‐industrialized participant populations	Grant, Ryan, Tigbe, & Granat, 2006; Kozey‐Keadle, Libertine, Lyden, Staudenmayer, & Freedson, 2011; Raichlen et al., 2020
Inclinometer (eg, activPAL)	Very high (≥95% agreement with direct observation)	Very high (inter‐device reliability >0.99)	Capable of differentiating sitting/lying from standing postures, small and discreet underneath clothing, possibility for continuous 24‐hour wear protocol	Adhesive can cause irritation to the wearer's skin	Very high (approximately $450 per unit)	High	Medium to high	Cannot differentiate from passive and active non‐ambulatory postures (eg, squatting, chair‐sitting), requires power (battery up to 2‐weeks) and data transfer, can be waterproofed (suitable for 24‐hour wear) depending on attachment method, requires pick‐up/postage	Edwardson et al., 2017; Grant et al., 2006; Kozey‐Keadle et al., 2011
Accelerometer (eg, Actigraph, GENEActiv, Axivity)									
Worn on thigh	Very high (≥90% agreement with direct observation)	Very high	Accurately detects posture and physical activity intensity	Devices were originally designed for wear on the hip or wrist; may be bulky and uncomfortable on the thigh	High (~$350 per unit)	High	Medium to high	Cannot differentiate from passive and active non‐ambulatory postures (eg, squatting, chair‐sitting), requires power (battery up to 2‐weeks) and data transfer, can be waterproofed (suitable for 24‐hour wear) depending on attachment method, requires pick‐up/postage	Edwardson et al., 2016; Pivarnik, Pfeiffer, Mudd, Biswas, & Montoye, 2016
Worn on wrist	Medium to high (depending on processing method)	Very high	Accurately detects physical activity intensity	Cannot differentiate between sitting and standing posture (depending on processing method)	High (~$350 per unit)	High	Medium to high	Requires power (battery up to 2‐weeks) and data transfer. Some (but not all) devices are waterproof. Requires pick‐up/postage.	Hildebrand, Hansen, van Hees, & Ekelund, 2017; Rowlands et al., 2014
Worn on hip	Low (58% agreement with direct observation)	Very high	Accurately detects physical activity intensity	Cannot differentiate between sitting and standing posture	High (~$350 per unit)	High	Medium to high	Requires power (battery up to 2‐weeks) and data transfer. Usually only used during waking hours and not in water. Requires pick‐up/postage.	Edwardson et al., 2016; Hildebrand et al., 2017
Wearable cameras	High (as long as the image coders are adequately trained to classify behaviors and the behaviors are visible and interpretable in the photos)	High (as long as the image coders are adequately trained to classify behaviors)	Gold standard for assessing context of sedentary behavior	Very intrusive to participants Very time‐intensive data analysis Technical limitations (eg, battery life, data storage) Complex ethical considerations	High	Very high	Very high	Complex ethical considerations regarding the people and behaviors that may be captured as the participant goes about everyday life, useful for understanding contextual information, requires access to memory cards, need recharging, can alter participant behavior	Doherty et al., 2013; Doherty et al., 2013; Kelly et al., 2013
Heart‐rate monitor (eg, Actiheart)	Low	Very high	Very good at assessing intensity of physical activity	Cannot detect posture, poor validity for sedentary time	Medium (~$50 per unit)	High	High	Sensitive to fluctuations in heart rate caused by non‐activity factors (eg, ambient temperature, stress), requires power	Judice, Santos, Hamilton, Sardinha, & Silva, 2015
Multi‐sensor devices (eg, SenseWear armband)	Low (correlation between activPAL and SenseWear: *r* = .37 (95%CI 0.13, 0.56)	Very high	Very good at assessing intensity of physical activity	Cannot detect posture, poor validity for sedentary time	High	High	High	Power and data storage considerations	Myers et al., 2017
Subjective measures									
Questionnaire ‐ Single item	Low (correlation with objective measures: *R* = .34 (95%CI 0.30, 0.39))	Variable (intra‐class correlations range from 0.41 to 0.86)	Easy to administer	Typically leads to under reporting, especially due to difficulty recalling total sitting time without prompts or possible social desirability bias	Very low	Low	Very low	Low burden, single question makes translation easy, may be subject to social desirability bias and difficulty in recalling overall sedentary time	Pooled validity estimate and reliability range from Bakker et al., 2020; See also Prince et al., 2020
Questionnaire—Domain‐based (composite)	Low For questionnaires with 2–9 items, *r* = .35 (95%CI 0.29, 0.41) compared to objective measures For questionnaires with ≥10 items *r* = .37 (95%CI 0.30, 0.43) compared to objective measures	Variable (intra‐class correlations range from 0.44 to 0.91)	Easy to administer, provides information on time spent in specific contexts, slightly better estimates of total sedentary time than single‐item questionnaires	Possible recall bias or social desirability bias, concurrent behaviors can lead to double‐counting, included behaviors may not be relevant outside of industrialized contexts	Very low	Low	Very low	Low burden, allows gathering of contextual information, may not be useful outside of northern, industrialized contexts, translation into different languages usually requires additional validation before use	Pooled validity estimate and reliability range from Bakker et al., 2020; See also Prince et al., 2020
Questionnaire—Domain‐based (time spent in specific behavior contexts)	Generally high	Variable	Easy to administer	Possible recall bias or social desirability bias	Very low	Low	Very low	Relevance of each behavior is culturally‐specific, only suitable for research questions interested in time spent in specific sedentary behavior contexts	Otten, Littenberg, & Harvey‐Berino, 2010; Wijndaele et al., 2014
Previous‐day recalls	High (correlation *ρ* > .75 compared to activPAL)	Medium (paucity of evidence, intra‐class correlation of .75)	Smaller chance of recall bias Allows inclusion of behaviors relevant to each participant without the imposition of pre‐specified behaviors as in a questionnaire	More labor‐intensive for both participants and researchers compared to questionnaires	Low	Medium	Medium	Flexible across languages and cultures as it is not bound by pre‐specified behaviors or specific languages (unlike questionnaires)	Gomersall, Pavey, Clark, Jasman, & Brown, 2015; Kohler et al., 2017; Matthews et al., 2013
Diaries	High (correlation *r* = .87)	Medium (intra‐class correlations range from .65 to .75)	Allows participants to list their activities rather than imposing a structure like in questionnaires. Low risk of recall bias if the diary is completed in a prospective manner.	Very time consuming, high likelihood that intensive self‐monitoring may lead to participants' reactivity, high likelihood of recall bias if participants complete the diary at the end of the monitoring period	Very low	High	High	Method can be tailored to the population (eg, pen‐and‐paper or electronic methods)	Bakker et al., 2020; Hart, Ainsworth, & Tudor‐Locke, 2011; Hart, McClain, & Tudor‐Locke, 2011
Ecological Momentary Assessment	Low (correlation *r* = .29 compared to activPAL and .16 compared to ActiGraph)	Unknown	Intermittent prompts allow insights into the context of behaviors, including where and with whom they are occurring	Burdensome to participants, prompts can disrupt the actual activity of interest, difficult to gather total sedentary time	Low to high, depending on whether devices (eg, mobile phones) need to be provided to participants by the researcher	Very high	Very high	Can be digital which is easiest to deliver but requires use of apps/mobile phones. Non‐digital is possible but more burdensome	Bakker et al., 2020; Degroote, DeSmet, De Bourdeaudhuij, Van Dyck, & Crombez, 2020; Maher, Rebar, & Dunton, 2018
Proxy‐report methods	Validity largely depends on the validity of the questionnaire being used	Reliability largely depends on the reliability of the questionnaire being used	Allows measurement of sedentary behavior in populations who might have difficulty with self‐report (eg, adults in need of special care, young children)	Disadvantages map onto the disadvantages of the kind of questionnaire being used	Very low	Low to medium	Very low	The suitability of the questionnaire for the cultural context must be considered (see above)	Matthews et al., 2011

### 2.1 Direct observation

Direct observation is a method by which a trained observer watches participants in the study and classifies their behavior according to predetermined criteria (eg, time spent in specific sedentary behavior contexts or time spent in specific postures) for a particular length of time. Direct observation is one of the oldest and most basic methods for measuring sedentary behavior, and is still used to this day for validation of novel sedentary behavior techniques (such as accelerometers; Giurgiu et al., 2020; Kozey‐Keadle et al., 2011) and for measurement in particular populations, such as hunter‐gatherers (Raichlen et al., 2020) and older inpatients with cognitive impairments (Belala, Maier, Heldmann, Schwenk, & Becker, 2019). Direct observation has strengths in the ability to measure posture, breaks in sedentary time, context for the behaviors, low equipment requirement, potential for high validity and reliability, and ability to distinguish between “non‐ambulatory” postures such as chair‐sitting, ground‐sitting, kneeling, and squatting. However, while it is intended to be objective, it does rely on the intra‐ and inter‐rater reliability of assessors and is significantly burdensome for everyone involved. This is due to the time commitment for continuous observation for the researcher and impact on privacy for the participant (Loprinzi & Cardinal, 2011). The continuous observation is also likely to change participant behavior, thereby reducing ecological validity. As such, it is more common and feasible to objectively measure sedentary time using device‐based measurements rather than direct observation.

### 2.2 Accelerometers and inclinometers

Accelerometers are a popular way of objectively measuring sedentary time in free‐living contexts, including in large‐scale population‐based studies (Matthews et al., 2008; Stamatakis, Davis, Stathi, & Hamer, 2012). Beginning in the 1990s, accelerometry was originally used in epidemiological studies for the objective measurement of physical activity (Freedson, Melanson, & Sirard, 1998; Troiano et al., 2008). Measurements of sedentary time were extrapolated from these accelerometry datasets through inference, with periods of non‐movement being interpreted as sedentary time (Healy et al., 2007; Matthews et al., 2008). With increasing recognition that it may be important to differentiate sitting from standing in the measurement of sedentary time (Owen, Healy, Matthews, & Dunstan, 2010; Sedentary Behavior Research Network, 2012), accelerometers specifically designed for the measurement of posture (“inclinometers”) became recognized for providing more accurate measures of time spent sitting. More recently, new ways of wearing accelerometers and processing their data to detect posture have been developed and are discussed below.

#### 2.2.1 Available devices and measurement details

**FIGURE 1:**
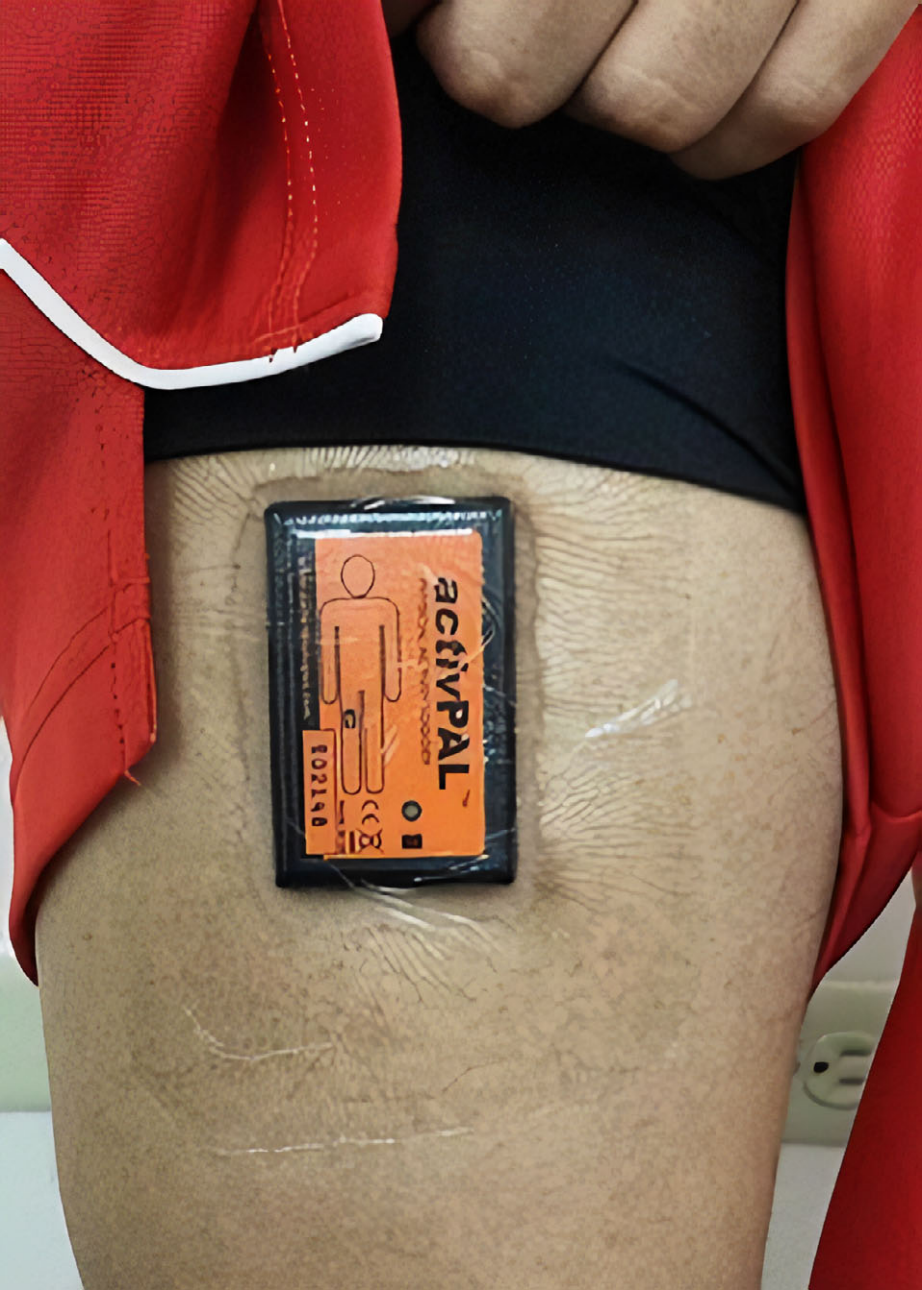
The activPAL v1 affixed to a participant's thigh using the 24‐hour waterproof attachment method

The activPAL inclinometer (PAL Technologies, Glasgow, UK; Figure 1) was the first inclinometer that was developed for large‐scale use and is now generally considered the “gold standard” for the objective measurement of sedentary time in free‐living contexts, with near‐perfect agreement against direct observation (Edwardson et al., 2016; Kozey‐Keadle et al., 2011). The activPAL is increasingly being used in large‐scale epidemiological and surveillance studies focused on the measurement of sedentary time (eg, Maastricht study [Schram et al., 2014]). The activPAL is a small device worn on the anterior midline of the thigh, affixed directly to the skin with adhesive and worn underneath clothing. It classifies sedentary time using a proprietary algorithm (Intelligent Activity Classification) when the thigh is stationary and within 20° of the horizontal plane (Bassett Jr. et al., 2014). By default, all classifications of sitting/lying posture are assigned a MET value of 1.25. The activPAL has been validated against direct observation for the measurement of total sedentary time, with ≥95% agreement in both laboratory‐based (Edwardson et al., 2016; Grant et al., 2006) and free‐living (Kozey‐Keadle et al., 2011; Lyden, Kozey Keadle, Staudenmayer, & Freedson, 2012) contexts. It has also been validated for the measurement of sit‐to‐stand transitions and breaks in sedentary time in free‐living contexts (Lyden et al., 2012).

Other tri‐axial (ie, can measure in three orthogonal directions) accelerometers are commonly used for the measurement of sedentary time, including the Actigraph (ActiGraph Ltd., Pensacola, FL), GENEActiv (ActivInsights, Newcastle upon Tyne, UK), and Axivity (Axivity Ltd., Newcastle, UK) devices. When worn on the thigh, these act as inclinometers similar to the activPAL. In this configuration, thigh‐worn accelerometers have high validity for the measurement of total sedentary time (Edwardson et al., 2016; Pivarnik et al., 2016) and breaks in sedentary time (Pivarnik et al., 2016) against direct observation as the criterion measure. However, these accelerometers are not typically worn on the thigh, as they were originally designed for wear on the hip (Matthews et al., 2008), and then the wrist (Troiano, McClain, Brychta, & Chen, 2014), reflecting their original purpose as a physical activity monitor.

When worn on the hip or wrist, accelerometers determine sedentary time based on periods of non‐movement without measuring posture. Historically, sedentary time has been determined when proprietary counts, usually counts per minute (cpm), were below a validated threshold, usually <100 cpm on the waist (Matthews et al., 2008) or <1853 cpm on the non‐dominant wrist (Koster et al., 2016). More recently, to avoid reliance on the “black box” nature of proprietary counts that differ between accelerometer manufacturers (Duncan et al., 2018), there is increasing use of raw gravitational acceleration in units of milli‐gravity (m*g*) collected via accelerometry. In these units, such as the GENEA acceleration sensor (STMicroelectronics, Geneva, Switzerland), sedentary time has been classed based on acceleration of <376 m*g* on the wrist or <96 m*g* on the waist when the accelerometer measurement frequency is 100 Hz (Esliger et al., 2011).

Regardless of whether counts or m*g* are used, wearing the accelerometer on the wrist or waist prevents detection of posture and therefore has low validity for the measurement of sedentary time (Edwardson et al., 2016; Hildebrand et al., 2017). However, there are methods for processing wrist‐worn accelerometry data that perform better for the measurement of sedentary time because they take the angle and rotation of the wrist into account. For example, the Sedentary Sphere (Rowlands et al., 2014), a Microsoft Excel worksheet developed for processing wrist‐worn accelerometer data, classifies sedentary time when movement is low and the angle of the arm is bent (ie, within 15° of the horizontal), suggesting the wearer is likely to be seated or reclining. The Sedentary Sphere has reasonable accuracy for detecting sedentary time in wrist‐worn configurations regardless of accelerometer brand (Rowlands et al., 2014; Rowlands et al., 2016), although it struggles in scenarios where the arm position does not match what would be expected by this arrangement; for example, standing at a standing desk would likely register as sitting because of the bent position of the arm. To our knowledge, no work has been done to evaluate the performance of this method for capturing variations of non‐ambulatory postures such as squatting or ground‐sitting. As this method relies on the position of the arm to identify posture, it would presumably classify postures such as squatting as sedentary time if the arm is bent, but this has not been empirically tested.

#### 2.2.2 Methodological considerations for the use of inclinometers and accelerometers

The inclinometer/accelerometer wear protocol is an important determinant of the accuracy of measurement. Over the past few years, with the shift toward using thigh‐ and wrist‐worn devices, it has become increasingly popular to use continuous wear protocols in which the device is worn 24‐hours per day (Edwardson et al., 2017; Troiano et al., 2014). Compared to waking‐wear protocols in which the device is removed for sleep and water‐based activities (a requirement of the waist‐worn configuration), participant compliance with continuous wear protocols is substantially higher because the wearer does not have to remember to put the device back on after a period of removal (Troiano et al., 2014). Continuous wear protocols can also reduce the risk of bias in measurement, both in terms of reactivity and wearing the device at selected (active) times of day, which is especially relevant for the measurement of sedentary time (Ryan et al., 2017). Regardless of whether a continuous or waking wear protocol is used, it is standard practice in the measurement of sedentary time to ask participants to wear the device for 7–8 days, with a dataset considered valid for analysis if the participant provides data for ≥10 hours on at least 4 days (Bellettiere et al., 2017).

A unique challenge in the accelerometer‐based measurement of sedentary time is distinguishing, from an acceleration and postural standpoint, “true” sedentary time from other behaviors that can resemble sedentary time, such as sleep and non‐wear time. There are a number of methods available to help identify and remove sleep and non‐wear from the dataset, including manual evaluations, participant diaries, and automated algorithms (see Edwardson et al., 2017). An example of a manual protocol is to assume that waking hours are 07:00 to 23:59; however, this technique may introduce large measurement error due to substantial interpersonal variability in sleep and wake times (Edwardson et al., 2017). Participant diaries are useful for capturing this variability in sleep schedules as participants record the time they go to bed and wake up each day (as well as when/whether they remove the device), but not all participants remember to complete and return their diaries. Automated algorithms are very useful for identifying periods of sleep and non‐wear because they do not impose assumptions about when sleep might occur; for example, an algorithm for activPAL data available in SAS or STATA identifies sleep as a bout of sitting/lying lasting ≥5 hours or the longest bout of sitting/lying (≥2 hours) per 24‐hour period, with additional rules to classify adjacent periods as sleep or awake (Winkler et al., 2016). Whenever these methods are used, it is advised to do visual checks (eg, producing heatmaps) to ensure that the algorithm's decisions appear sensible and apply manual corrections if necessary (Edwardson et al., 2017). A toolkit by Samson (2020) regarding the measurement of sleep may be the best resource for those looking to measure and remove sleep time from sedentary behavior analyses.

Throughout the process of selecting a device and devising the wear protocol, it is essential to consider practicalities of wear for the participant. One of these considerations is how discreet the device is; if it is bulky or conspicuous, participants may take it off in some situations (see Pollard & Guell, 2012). The activPAL is generally inconspicuous on the thigh (particularly the newer activPAL3 micro and activPAL4 models), the wrist‐worn GENEActiv and Axivity resemble sport watches, and the Actigraph is comparatively large and is colored brightly red so may be less appealing to participants. Another issue is that, in continuous wear protocols in which the device is attached with adhesive to the skin (eg, with the activPAL), participants can develop skin irritation (Edwardson et al., 2017; Wagnild et al., 2019). This can be mitigated by forewarning participants that this may occur and helping them to pre‐empt it by moving the device to the same spot on the other leg (with extra adhesive provided). Lastly, as with many electronic devices, attention must be paid to power requirements, waterproofing, and the potential for damage during data‐collection processes.

### 2.3 Wearable cameras

Wearable cameras (eg, SenseCam, Autographer) are among the most innovative technological tools available for objectively measuring a range of lifestyle behaviors, including sedentary time. The camera is usually worn on a lanyard around the neck and captures time‐stamped images approximately once every 20 seconds as the wearer goes about their everyday life (Doherty, Hodges, et al., 2013; Doherty, Kelly, et al., 2013). The main benefit of using wearable cameras in this way is that they combine an objective measurement of time spent in various sedentary behaviors and physical activities with contextual information about the specific kind of activity performed, where, and with whom. This may be especially useful when trying to measure contextual information about time spent in sedentary behaviors, particularly to capture concurrent behaviors such as using a mobile device while watching TV, or watching TV while eating snacks and meals (Gemming, Doherty, Utter, Shields, & Ni Mhurchu, 2015).

The two main drawbacks to using wearable cameras are ethical/privacy considerations and researcher burden. Some ethical issues are mitigated by giving wearers the power to stop recording at any time and to delete, at the end of the wear period, any photographs that they wish the researchers not to see (Kelly et al., 2013). However, other ethical issues are more complex, such as how to obtain consent from third parties captured in the images (particularly those who live with the participant) and what the research team should do in the event that illegal behavior is captured (Kelly et al., 2013). In terms of researcher burden, the volume of images generated from wearable cameras is substantial—for example, 364 000‐500 000 images can be generated from a sample of 40‐50 participants providing 3‐4 days of data (Kelly et al., 2013; Kerr et al., 2013), which the researcher must then annotate using a coding framework that suits the particular research question.

### 2.4 Heart rate monitoring and combined heart rate and movement monitoring

Heart rate monitoring has been used as a measurement of physical activity for decades (Spurr et al., 1988; Wareham, Hennings, Prentice, & Day, 1997). Typically, heart rate has been used to estimate total energy expenditure or time spent at higher physical activity intensities (eg, MVPA) based on individually‐calibrated thresholds that differentiate rest from higher‐intensity movement (flex‐HR method; [Spurr et al., 1988]). Since then, heart rate monitoring has also been used for the measurement of sedentary time, defined as daily time spent below the flex‐HR threshold (Helmerhorst, Wijndaele, Brage, Wareham, & Ekelund, 2009). The validity of heart rate monitoring is low at lesser movement intensities, as it is particularly susceptible to fluctuations caused by non‐movement factors such as ambient temperature and stress levels (Livingstone, 1997). While heart rate has been shown to be significantly higher while standing compared to sitting in controlled laboratory conditions (Gao et al., 2017; Judice et al., 2015), it is unclear whether heart rate monitoring can accurately differentiate postures in uncontrolled free‐living conditions.

Devices are available that combine heart rate monitoring with accelerometry (eg, Actiheart, Actitrainer). The main feature of this combined method is to be able to identify both periods of non‐movement (based on accelerometry/inclinometry) and intensity (based on heart rate) to determine energy expenditure. This has the potential to improve accelerometer‐only measures of sedentary time because it could theoretically differentiate seated physical activities (eg, weightlifting, cycling) from sedentary time by detecting the increased heart rate associated with these seated exercise activities. However, in practice, these devices have demonstrated poor validity for the measurement of sedentary time. For example, in one free‐living validation study, the Actiheart underestimated sedentary time by 156 minutes per day and overestimated the number of daily breaks in sedentary time by 235 per day (Judice et al., 2015).

### 2.5 Multi‐sensor monitors

There are some devices that use multiple sensors to classify physical behaviors, including sedentary time. The benefit of these devices could be the potential to capture all aspects of the sedentary behavior definition, including energy expenditure of specific behaviors while seated. For example, some seated behaviors such as intense video gaming have been found to be expending energy in excess of 1.5 METs (Mansoubi et al., 2015) which do not technically fit within the definition of sedentary behavior. Multi‐sensor devices can be combinations of accelerometers and physiological sensors; for example, devices such as the SenseWear armband measure acceleration, heat flux, galvanic skin response, skin temperature, and ambient temperature to estimate energy expenditure using proprietary algorithms. As such, sedentary time is classified as time spent with energy expenditure below 1.5 METs. The SenseWear is not able to detect posture, and, as a result, tends to classify both standing and sitting as sedentary (where METs are <1.5), resulting in significant overestimations of sedentary time (Myers et al., 2017).

Other devices use multiple accelerometers at various attachment points. For example, the Intelligent Device for Energy Expenditure and Physical Activity (IDEEA) has five sensors taped to the soles of both feet, on both thighs, and on the chest, that connect to a central processing unit clipped at the waist. A neural network integrates the data from all of the sensors to classify the body's position into one of 32 validated postures (Zhang, Werner, Sun, Pi‐Sunyer, & Boozer, 2003). The IDEEA has demonstrated high validity for the measurement of sedentary time and the ability to distinguish between seated and standing postures in both laboratory and free‐living settings (Hart, McClain, & Tudor‐Locke, 2011; Jiang & Larson, 2013; Zhang et al., 2003), but the multiple wired sensors may be unmanageable for participants over multiple days (Welk, McClain, Eisenmann, & Wickel, 2007).

### 2.6 Combining objective methods

In addition to multi‐sensor devices, one could simply combine objective methods to address the shortcomings of each approach. For example, the SenseWear armband (which can determine energy expenditure but not posture) and the activPAL (which can determine posture but not energy expenditure) have been used simultaneously to create an integrated measure of sedentary time that accounts for both posture and energy expenditure (Myers et al., 2018). Similarly, wearable cameras and Actigraph accelerometers have been used in conjunction to assess the context and intensity of daily physical activity (Kerr et al., 2013). While such approaches may improve the measurement of sedentary time, it is unclear whether the additional information justifies the participant and researcher burden of using multiple monitors. The use of multiple monitors also comes with added costs to the research team in terms of the devices, consumables for attachment (eg, adhesives), and data processing considerations. It is therefore worth considering carefully the trade‐off between (often slight) increases in measurement precision and increases in research and participant burden.

### 2.7 Special considerations for field use

It is worth noting that the validity of inclinometers and thigh‐worn accelerometers has been established within the context of northern industrialized populations, for whom the horizontal position of the thigh is likely to correlate with a seated or reclined posture. This may result in misclassifications in populations where postures such as squatting and kneeling, which are not classically considered sedentary behaviors, are common. For example, data from the Hadza show that approximately 30% of time that the activPAL classifies as sitting/lying was actually spent squatting or kneeling (Raichlen et al., 2020). To differentiate between “active” and “passive” postures in these kinds of contexts, it may be particularly useful to combine an inclinometer with another method, such as direct observation, a wearable camera, or electromyographic shorts (Tikkanen et al., 2013).

## 3 SUBJECTIVE MEASUREMENT OF SEDENTARY BEHAVIOR

There are a substantial number of subjective (self‐reported) methods for measuring sedentary behavior, including self‐report questionnaires, previous‐day recalls (PDR), diaries, and ecological momentary assessments (EMAs; Table 1). Due to the flexibility around what kind of questions can be asked, subjective techniques can theoretically capture the purpose, environment, posture, social context, associated behavior, status, time, and type of sedentary behavior (Rivière, Aubert, Omorou, Ainsworth, & Vuillemin, 2018). While objective measurements are typically validated against direct observation, subjective measures are often compared against objective ones. The types of subjective measures currently available to researchers are discussed below and, where applicable, their validity is discussed in relation to the activPAL, considered here as the gold‐standard for the measurement of free‐living sedentary time.

### 3.1 Self‐report questionnaires

#### 3.1.1 Total assessment or single item questionnaires

“Total assessment” or “single item” questionnaires ask participants to retrospectively estimate and report their total sitting time over a specified recall period. For example, the International Physical Activity Questionnaire (IPAQ) Short Form (Craig et al., 2003) asks “During the last 7 days, how much time did you spent sitting on a week day?” with respondents providing a response in terms of hours and minutes per day. Single‐item measurements of sedentary time have been found to substantially underestimate sedentary time; the IPAQ Short Form, for example, has been shown to underestimate total sedentary time by 161.7 (95%CI 97.0, 226.4) minutes per day vs device‐assessed sedentary time (Prince et al., 2020). This measurement error is not specific to the IPAQ; a recent meta‐analysis pooling the criterion validity of all available single‐item questionnaires showed weak correlations with objective measures of sedentary time (*r* = .34 [95%CI 0.30, 0.39]) (Bakker et al., 2020). This discrepancy between measures may be attributable to social desirability bias or may simply reflect difficulty in estimating the total amount of time spent sitting, especially as sedentary time can be accumulated through both structured (eg, watching a 30‐minute TV show) and unstructured (eg, sitting in a waiting room) activities.

#### 3.1.2 Domain‐based questionnaires

##### Composite measures of total sedentary time

Domain‐based questionnaires are often used to estimate total sedentary time by summing up time spent in specific sedentary behavior contexts. For example, the Sedentary Behaviour Questionnaire (SBQ; [Rosenberg et al., 2010]) asks about time spent in nine sedentary behavior contexts (watching TV, playing computer/video games, sitting while listening to music, sitting and talking on the phone, doing office work or paperwork, reading, playing a musical instrument, doing arts and crafts, and driving/riding in a car, bus, or train) on both a typical week and weekend day. The responses across these behaviors are then summed to estimate daily or weekly total sedentary time. Composite measures of sedentary time tend to produce more accurate estimates of total sedentary time than single‐item recalls (Prince et al., 2020), perhaps because it is easier for participants to recall time spent performing specific activities rather than total time spent sitting (Healy et al., 2011). Despite this, multi‐item questionnaires still tend to underestimate total sedentary time (Prince et al., 2020) and only weakly correlate with objective measures of sedentary time (*r* = .37 [95%CI 0.30, 0.43]) (Bakker et al., 2020).

One of the key challenges unique to composite measures of total sedentary time is the possibility of double‐counting behaviors that occur simultaneously. For example, in the United States, the questionnaire developed for the National Health and Nutrition Examination Survey (NHANES) asks participants about daily time spent watching TV and time spent using a computer or tablet over the past 30 days, and these two questions are sometimes summed together as a measure of “screen time” (eg, Madhav, Sherchand, & Sherchan, 2017). Although some questionnaires try to alert participants to only report the “main” behavior they were engaged in at a given time, there is a possibility for the occurrence of double‐counting if a participant has multi‐tasked (ie, browsed on a tablet while watching TV) but reported the behaviors separately. Additionally, if the participant is not keenly aware of the postural requirements of the questionnaire, it may be the case that people are standing while doing some of the behaviors that are asked about, and these may be incorrectly counted toward sedentariness. Another key issue in the use of composite measures is that the estimation of total sedentary time depends on the relevance of the specific behaviors asked about to the participant population. As domain‐specific questionnaires for capturing time spent in sedentary behaviors have been developed in northern, industrialized contexts, they may not be suitable for use in different cultural contexts. This means that researchers targeting non‐Western populations may have to develop and validate new domain‐specific questionnaires against objective devices or direct observation prior to beginning their study to ensure that key, relevant behaviors are included. Even within industrialized contexts, many questionnaires such as the Measure of Older Adults Sedentary Time (Gardiner et al., 2011) focus on “leisure time” activities but miss key behaviors such as eating, which is typically seated and occupies a large amount of the day (Aunger et al., 2020). It is also important to note that questionnaires have generally been written and validated in English and may require specific validation for use in other languages. Additionally, they require relatively frequent updating to ensure culture‐appropriate and relevant changes in technology and behavioral norms (ie, increases in smartphone use) are captured in the questionnaire.

##### Measures of time spent in specific sedentary behaviors

Domain‐based questionnaires can also be used to ascertain time spent in specific sedentary behavior(s) of interest, not just total sedentary time. For example, the health outcomes associated with time spent watching television (Grøntved & Hu, 2011) or time spent sitting at work (van Uffelen et al., 2010) have long been of research interest. The validity of measuring time spent in single behaviors is generally high. For example, self‐reported time spent watching television has been shown to have high agreement (*ρ* = .84, *P* < .001) with objective measurements of television time (Otten et al., 2010; Wijndaele et al., 2014), and self‐reported time spent sitting at work shows good agreement (*ρ* = .63, *P* < .001) with objective measures (Wijndaele et al., 2014).

There are two critical considerations when using domain‐based questions in this way. First, questions about time spent in specific sedentary behaviors must be individually validated. Single questions cannot be extracted from validated questionnaires and used on their own as the validity of a questionnaire specifically applies to the questionnaire as a whole. Second, the measurement of time spent in a specific sedentary behavior cannot be extrapolated as an estimate of total sedentary time. For example, television time is often used as a proxy for total sedentary time and this is problematic because these two constructs are only weakly correlated (Clark et al., 2011; Clark et al., 2015; Wagnild & Pollard, 2020).

#### 3.1.3 Previous‐day recalls

PDRs are useful tools for capturing detailed information about sedentary behavior that took place on the previous day. Using a format similar to the 24‐hour Physical Activity Recall (Calabro et al., 2009), PDRs use semi‐structured interviews to ask each participant about their activities on the previous day in a chronological format. For example, the participant is asked, starting from when they woke up, about what they did on the previous morning. Any activities that lasted at least 5 minutes are recorded (with their duration); this process continues until the entire day's activities have been recalled. Time spent in activities that took place while sitting, reclining, or lying down are then summed to estimate total sedentary time on the previous day. Total sedentary time measured by PDR has been shown to be strongly correlated (*ρ* > .75) with activPAL‐measured sedentary time, although PDR provided a slight overestimate (Gomersall et al., 2015; Matthews et al., 2013).

The PDR approach has several strengths. Unlike questionnaires, PDR approaches do not impose assumptions about the kinds of behaviors in which participants are likely to engage. This may be particularly useful for measuring sedentary behavior in cultural contexts in which sources of sedentary time differ from the kinds of “Western” behaviors included in questionnaires. They can also be delivered in the native languages of the population of interest. PDR approaches are also able to capture sedentary behaviors that are common but often excluded from questionnaires, such as sitting to eat a meal (Aunger et al., 2020). The semi‐structured interview nature of PDR also lends itself to clarifications to understand the postures associated with each activity the participant reports.

#### 3.1.4 Diaries and ecological momentary assessments

Diaries allow participants to record their daily activities, including sedentary behaviors, in a prospective manner. For example, the Bouchard activity record typically uses a pen‐and‐paper approach and asks participants to record their daily activities in 15‐minute intervals spanning the entire 24‐hour day. From this, by summing the 15‐minute blocks in which a sedentary behavior was recorded, total sedentary time can be estimated. The Bouchard activity record has been shown to have a very strong correlation with activPAL‐measured sedentary time (*r* = .87, *P* < .05) (Hart, Ainsworth, & Tudor‐Locke, 2011).

Ecological momentary assessments (EMAs; Shiffman, Stone, & Hufford, 2008; Stone & Shiffman, 1994) are also real‐time, prospective assessments of behavior, involving multiple prompts sent to the participant (usually via a smartphone or similar device) to report their current behavior at various intervals throughout the day. EMAs are typically employed for a capture period of 4‐8 days and prompt intervals can range from 15 minutes to 2 hours (Romanzini et al., 2019). The EMA approach does not rely on recall and offers responsive opportunities to explore contextual information by including additional questions such as where and with whom the reported behavior is occurring. The validity of EMAs for measuring total sedentary time is low, with a weak correlation (*r* = .29) compared to activPAL measures of sedentary time (Maher et al., 2018). However, EMAs can offer unique insights into co‐occurrences or contexts of sedentary behavior, such as whether snacking is more likely to happen while watching TV compared to other behaviors (Ghosh Roy, Jones, Martyn‐Nemeth, & Zenk, 2019).

The key strength of diaries and EMAs is that the recording of behaviors in real‐time reduces the likelihood of recall bias. However, while diaries are meant to be filled out in real‐time, it is possible that participants may forget to complete the diary as they go and fill it in at the end of the study period using recalled estimates. Both diaries and EMAs also come with a substantial burden, both on the part of the participant and the researcher due to requirements for data cleaning and entry. With such intensive self‐monitoring involved in these approaches, there is also a particular risk of the “Hawthorne effect” whereby participants modify their activities in response to being monitored. More frequent intervals of sampling have also been found to cause people to avoid partaking in physical activity so that they can answer the prompts, causing an increase in sedentary behavior (Maher et al., 2018; Rouse & Biddle, 2010). Additionally, while EMAs allow freedom to ask follow‐up questions to gather contextual information related to (or co‐occurring with) the sedentary behavior of interest, the criterion validity of such probes has not yet been established (Degroote et al., 2020). Due to the reliance on technology for many EMA techniques, limitations inherent to objective measures for field use (ie, electricity requirements) must also be considered if EMAs are to be employed.

#### 3.1.5 Proxy‐report methods

Proxy‐reporting techniques can be used in populations, such as young children or adults in need of special care, where their own self‐report may not be accurate or reliable (Atkin et al., 2012; Hardy et al., 2013). These proxy techniques are typically used for children or adults with intellectual disabilities (Melville et al., 2017), and can take the form of single‐item “total” assessment of sedentary time (Wen, Van der Ploeg, Kite, Cashmore, & Rissel, 2010), diaries (Melville et al., 2017), or domain‐based techniques (Lubans et al., 2011; Salmon, Campbell, & Crawford, 2006). One example is the IPAQ proxy respondent version, which has been adapted from a 7‐day recall to a diary‐based measure which is completed by a carer. The validity of proxy‐report methods tends to be variable but low, in line with the validity of the specific subjective tool (eg, questionnaire, diary) that is being completed by proxy.

## 4 COMBINING OBJECTIVE AND SUBJECTIVE METHODS

From a measurement perspective, objective and subjective methods for measuring sedentary time/behavior have different strengths and weaknesses. Objective measurements (particularly using thigh‐worn devices) have the highest validity and are sufficient on their own for studies where the aim is to quantify any combination of total sedentary time, breaks in sedentary time, or sit‐to‐stand transitions. However, with the exception of wearable cameras, objective measures do not provide any information about the specific behaviors or contexts that contribute to total sedentary time. Subjective measurements, on the other hand, tend to have comparatively low validity but can offer rich contextual information about when and where sedentary behavior is occurring. If the aim of a study is not to quantify total sedentary time but to understand how and where sedentary behavior is occurring, subjective methods are preferable.

Objective and subjective methods capture different, and potentially complementary, aspects of sedentary behavior, and using them together can provide a more comprehensive measure of sedentary behavior than either one of them alone. Troiano et al. (2012) describe different ways in which these approaches can be used together: combined, linked, or integrated. The combined approach, used often in epidemiological studies with health‐related outcomes, includes the use of both objective and subjective measurements to explore differences in effects and to understand possible sources of variation in total sedentary time (eg, Stamatakis, Davis, et al., 2012; Wagnild et al., 2019; Wagnild & Pollard, 2020; Wennman, Vasankari, & Borodulin, 2016). The linked approach involves, for example, the use of logs or diaries concurrently with accelerometry to match behaviors with accelerometer data through matching time‐stamps (eg, Edwardson et al., 2018). Finally, the integrated approach draws on both objective and subjective methods to substantiate and contextualize each other, for example through the combination of accelerometry and EMA (eg, Liao, Intille, & Dunton, 2015).

Objective and subjective methods can also be used together to “calibrate” subjective methods to improve their validity. For example, several validation studies (Coenen, Mathiassen, van der Beek, & Hallman, 2020; Hallman, Mathiassen, van der Beek, Jackson, & Coenen, 2019; Metcalf et al., 2018; Welk, Beyler, Kim, & Matthews, 2017) have asked participants to wear an accelerometer for a period of time and then, at the end of the wear period, complete a questionnaire or past‐day recall referring to sedentary behaviors during that period. A range of statistical methods (based on linear regression) were then used to correct the biases in subjective measurement against accelerometry, resulting in a calibrated version of the questionnaire with lower measurement error. These calibrated values can then be used to correct subsequent (repeated) subjective measurements in same population without requiring additional accelerometry measurements. However, use of these techniques requires significant statistical expertise. Interestingly, Metcalf et al. (2018) have demonstrated the possibility of calibrating subjective measures (from the Global Physical Activity Questionnaire) in one sample and applying the calibrated values to an independent sample. This creates possibilities for improving the validity of subjective measurements in the future.

## 5 OVERALL RECOMMENDATIONS AND CONCLUSION

Objective measures, particularly inclinometers or thigh‐worn accelerometers, have the highest validity for the measurement of sedentary time, but they cannot determine what kinds of sedentary behaviors are taking place. Subjective measures, particularly PDRs and diaries, are effective for capturing time spent in various sedentary behaviors but have comparatively low validity for measuring total sedentary time. Wherever resource allows, researchers should consider the use of both objective and subjective methods to capture total sedentary time, the pattern of its accumulation, as well as the behaviors that are occurring during these periods.

It is essential when selecting measurement approaches to consider the appropriateness and relevance of the method for the population of interest. As methods for measuring sedentary behavior have been developed within industrialized contexts, their validity is likely to differ outside of these settings. This is especially pertinent for inclinometers, where the horizontal position of the thigh is assumed to correlate with a sitting/reclining posture (thus including non‐sedentary postures such as squatting in estimates of sedentary time), and for domain‐based questionnaires, where time spent in specific behaviors that are common in Western contexts are measured.

## AUTHOR CONTRIBUTIONS

**Justin Aunger and Janelle Wagnild**: Conceptualization; formal analysis; methodology; writing‐original draft; writing‐review and editing.

## CONFLICT OF INTEREST

The authors declare no potential conflict of interest.
